# Hypernatremia-Associated Catatonia in a Renal Transplant Recipient: A Diagnostic Challenge

**DOI:** 10.51894/001c.165957

**Published:** 2026-07-31

**Authors:** Razan Mohanna, Kamal Khalil, Khabbab Alnimer

**Affiliations:** 1 DMC - Sinai Grace

## 103

Catatonia is a neuropsychiatric syndrome characterized by motor, behavioral, and affective abnormalities and is most commonly associated with psychiatric illness, though medical etiologies are increasingly recognized. We report the case of a 77-year-old female renal transplant recipient who presented with profound altered mental status, hypernatremia, and tacrolimus toxicity, complicated by a catatonia-like presentation. Despite correction of metabolic derangements, she exhibited persistent episodes of mutism, stupor, and unresponsiveness. A Bush–Francis Catatonia Rating Scale score of 18 supported the diagnosis of catatonia. Intravenous lorazepam challenge testing produced transient but reproducible clinical improvement, confirming catatonia secondary to a medical etiology. This case highlights the importance of recognizing catatonia in medically ill patients, particularly in the setting of metabolic disturbances and immunosuppressant toxicity, to avoid diagnostic delay and unnecessary investigations.

